# Pseudoarthrosis of the ilium after periacetabular osteotomy that was treated by cemented total hip arthroplasty: a case report

**DOI:** 10.1186/s13256-016-0899-2

**Published:** 2016-05-06

**Authors:** Arihiko Kanaji, Toru Nishiwaki, Akihito Oya, Kazuyuki Maehara, Hideki Maehara, Teruyo Oishi, Harumoto Yamada, Yasunori Suda, Masaya Nakamura, Morio Matsumoto

**Affiliations:** 1 Department of Orthopaedic Surgery, Keio University School of Medicine, 35 Shinanomachi, Shinjuku, Tokyo 160-8582 Japan; 2 Department of Orthopaedic Surgery, Fujita Health University, 1-98 Dengakugakubo, Kutsukake, Toyoake, Aichi 470-1192 Japan; 3 Maehara Surgery, Orthopedic Surgery, Pediatrics, 16-1 Nishinokaido, Ano-cho, Toyoake, Aichi 470-1144 Japan

**Keywords:** Total hip arthroplasty, Periacetabular osteotomy, Pseudoarthrosis

## Abstract

**Background:**

Preserving the hip joint to delay arthroplasty for patients with acetabular dysplasia-associated early-stage osteoarthritis has become more common, and several surgical procedures have demonstrated pain relief and improved hip joint function. Periacetabular osteotomy, one of the joint-preserving surgical procedures of the hip, provides favorable outcomes, although there are no reports of total hip arthroplasty being used to treat pseudoarthrosis of the periacetabular osteotomy segment. Therefore, we report a case of pseudoarthrosis in the osteotomy segment after periacetabular osteotomy. The patient was treated using modified total hip arthroplasty and achieved a favorable short-term outcome.

**Case presentation:**

A 62-year-old Japanese woman was diagnosed with bilateral acetabular dysplasia at the age of 50 years, and underwent right and left periacetabular osteotomy at the ages of 52 and 55 years, respectively. When she was 61-years old, she experienced repeated episodes of left coxalgia during walking, with increasing pain at rest, and subsequently visited our department. Plain radiography and computed tomography of her left hip joint confirmed pseudoarthrosis of the periacetabular osteotomy segment. In addition, narrowing of her left hip joint space was observed, which indicated advanced osteoarthritis of the hip. Therefore, she underwent left total hip arthroplasty when she was 62-years old. During the surgery, fibrous fusion of the periacetabular osteotomy segment was confirmed via fluoroscopy, although no abnormal mobility was observed. Thus, the osteotomy segment was fixed with one absorbable screw and two bone pegs (which were prepared using allogeneic bone), and the acetabular cup was fixed using cement. Her postoperative course was generally favorable and bone fusion of the periacetabular osteotomy segment was confirmed at 3 years and 6 months after surgery. Her modified Harris hip score was 43 before the surgery and had improved to 90 at the final follow-up.

**Conclusions:**

Modified total hip arthroplasty was successfully used to treat osteoarthritis of the hip and pseudoarthrosis of the periacetabular osteotomy segment. This procedure achieved pseudoarthrosis region bone fusion and a favorable postoperative outcome.

## Background

During developmental dysplasia of the hip (DDH), the acetabular side exhibits a sharp and shallow articular surface, while the femoral side exhibits bone morphological abnormalities, such as coxa valga and excessive femoral neck anteversion. Unfortunately, acetabular dysplasia can cause the acetabulum to provide poor coverage of the anterior to the upper regions of the femoral head, which allows osteoarthritis of the hip to progress for an extended period. Thus, joint-preserving surgery, such as periacetabular osteotomy (PAO), should be considered for young patients with acetabular dysplasia-associated pre-stage and early-stage osteoarthritis of the hip.

Among young patients with pre-advanced stage DDH, many reports have described favorable postoperative and long-term outcomes after PAO [1]. However, the outcomes in middle-aged and older patients (>50 years old) after joint-preserving surgery are poor in some cases. In these cases, the selection of a surgical method is controversial, because of the recently improved survival rate after total hip arthroplasty (THA) [2–5]. In the present case, we encountered a patient who had developed pseudoarthrosis in her osteotomy segment after PAO, and we successfully treated her via modified THA.

## Case presentation

A 62-year-old Japanese woman (height 149 cm, body weight 72 kg, body mass index 32.4 kg/m^2^) had received plaster treatment for DDH at birth, and observation of her clinical course without treatment was performed throughout her infancy and while she attended school. However, non-induced bilateral coxalgia appeared during walking at the approximate age of 45 years, and she visited a physician when she was 50-years old. She was diagnosed with bilateral acetabular dysplasia, underwent right PAO when she was 52-years old, and then underwent left PAO when she was 55-years old (at another hospital). However, pseudoarthrosis of her left PAO segment was diagnosed at 1 year after surgery, although only observation of her clinical course was performed because she did not experience any pain.

When she was 61-years old, left coxalgia during walking reappeared, as well as increasing pain at rest, and she visited our department. Plain radiography and computed tomography (CT) of her left hip joint confirmed pseudoarthrosis of the PAO segment. In addition, narrowing of her left hip joint space was observed, indicating advanced osteoarthritis of her hip, and she was admitted to undergo left THA during May 2010.

### Status at the first examination

**Fig. 1:**
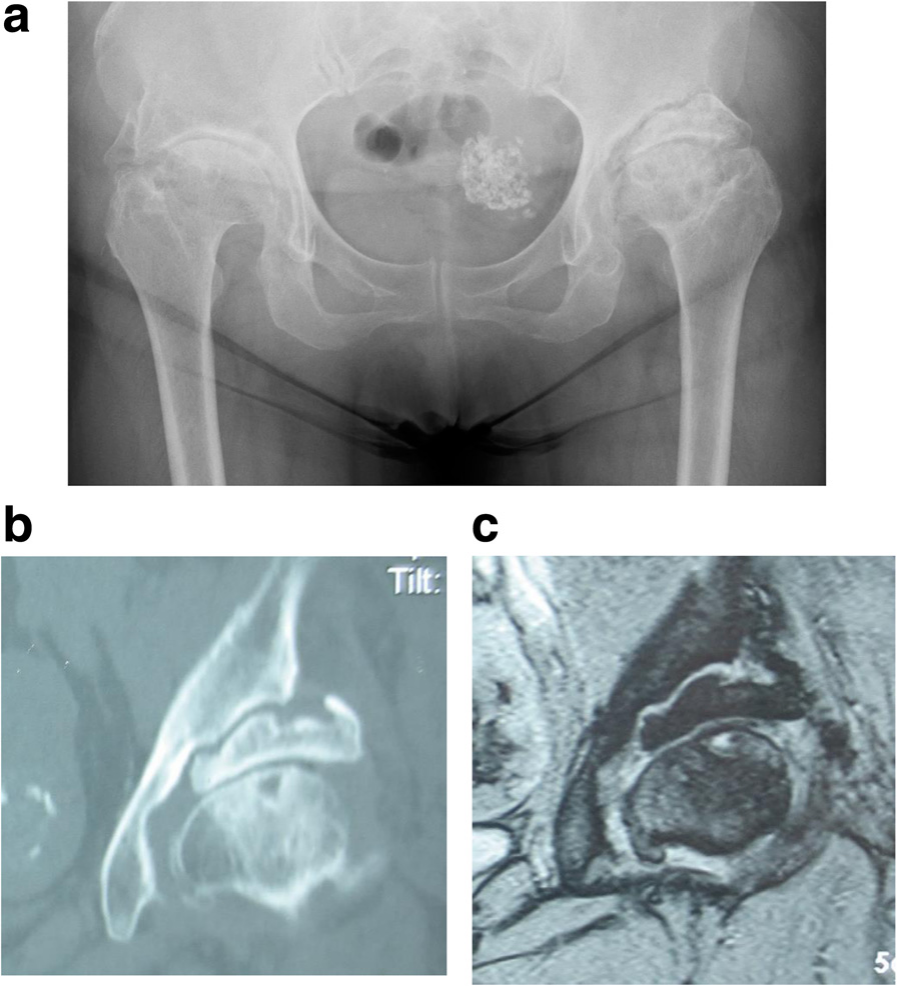
Imaging findings from the first examination. **a** Plain radiography of the bilateral hip joints reveals no joint space narrowing in the right hip joint. However, joint space narrowing and pseudoarthrosis of the periacetabular osteotomy segment is visible in the left hip joint. **b** Computed tomography of the bilateral hip joints reveals no bone fusion at the left segment. **c** Magnetic resonance imaging of the bilateral hip joints reveals pseudoarthrosis at the periacetabular osteotomy mobile bone segment of the left hip joint, with synovial fluid retention. However, no distal segment necrosis is visible

Claudication and Trendelenburg’s sign were observed during single crutch walking. There was no leg length discrepancy, although the preoperative range of motion of her left hip joint was 90° flexion, 0° extension, 10° abduction, 10° adduction, 20° external rotation, and 10° internal rotation. Radiography of her bilateral hip joints revealed no narrowing in her right hip joint, although joint space narrowing and pseudoarthrosis of the PAO segment were observed in her left hip joint (Fig. 1a). CT and magnetic resonance imaging (MRI) did not reveal bone fusion of the left PAO segment; however, osteonecrosis or bony cystic lesion of the PAO mobile bone segment was not observed in the MRI (Fig. 1b, c).

### Surgical findings

**Fig. 2:**
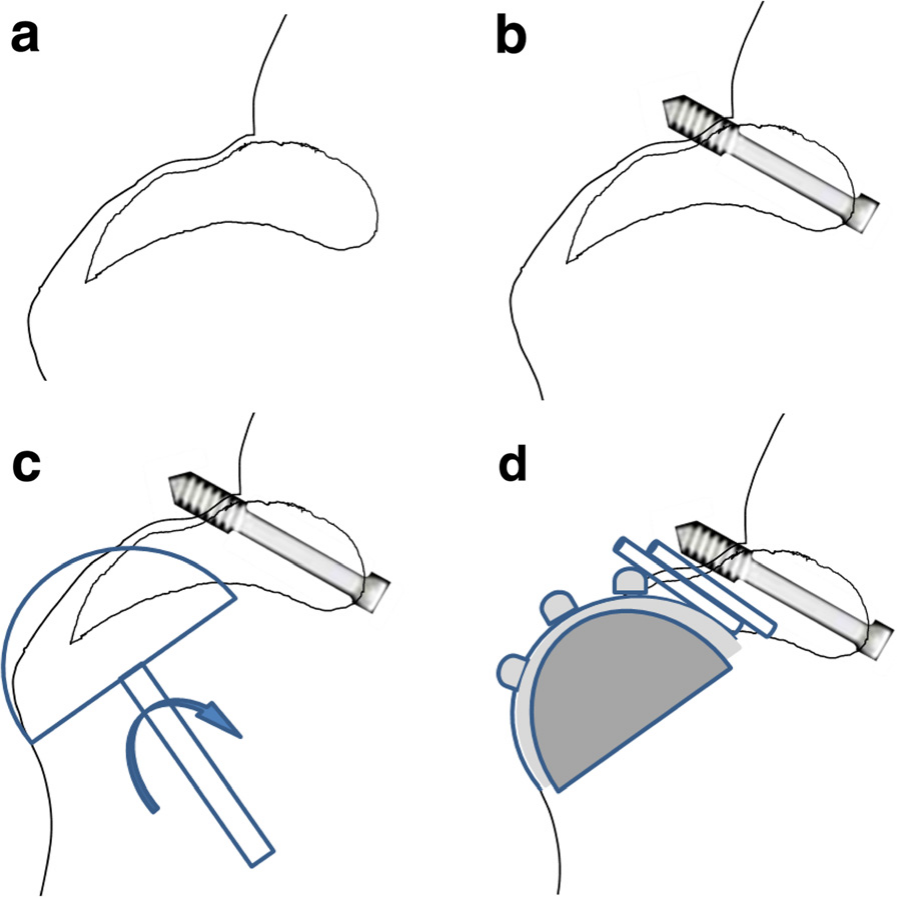
Surgical findings (acetabular side). **a** The periacetabular osteotomy segment had not achieved osseous fusion. Only fibrous fusion is visible, which raised concerns regarding intraoperative exacerbation of the osteotomy segment’s instability and poor initial fixation of the acetabular cup. **b** As fibrous fusion of the pseudoarthrosis region is visible, and as dissection can increase surgical stress, the periacetabular mobile bone segment was fixed with one absorbable screw. **c** To provide favorable cup fixation, drilling was used to expose the native bone and the cup was placed. Acetabular reaming was applied slightly above the original acetabular level, and the pseudoarthrosis region was stabilized using fixation and an absorbable screw. **d** To provide bone fusion at the pseudoarthrosis region, two bone pegs were prepared with autologous and allogeneic bone, and were subsequently transplanted

During May 2010, a left THA was performed via the Hardinge approach, with the patient in a supine position, using the C-Prominent cemented THA system (Nakashima Medical Co. Ltd.). After exposing the region around her hip joint, fibrous fusion of the PAO segment was confirmed via fluoroscopy, although no abnormal mobility was observed (Fig. 2a). Thus, the osteotomy segment was fixed using one absorbable screw, although we did not perform dissection to prevent hemorrhage (Fig. 2b). Acetabular reaming was applied 3 cm above the original acetabular level in order to achieve sufficient cup stability with host bones (Fig. 2c), and the residual mobile bone segment was fixed using two bone pegs that were prepared using allogeneic bone and the excised femoral head (Fig. 2d). However, it was unclear whether favorable bone ingrowth fixation could be achieved via cementless fixation, because the quality of the PAO mobile bone segment was unclear. Thus, the cup was fixed using cement. The operative time was 3 hours and 31 minutes, and the intraoperative blood loss was 694 mL. Postoperative plain radiography of her bilateral hip joints revealed that the inclination angle of her acetabular cup was 46°, that the cup-center-edge (CE) angle was 27°, that the femoral stem was inserted at the neutral position, and that the alignment of the acetabular cup and stem was favorable.

### Postoperative course

**Fig. 3:**
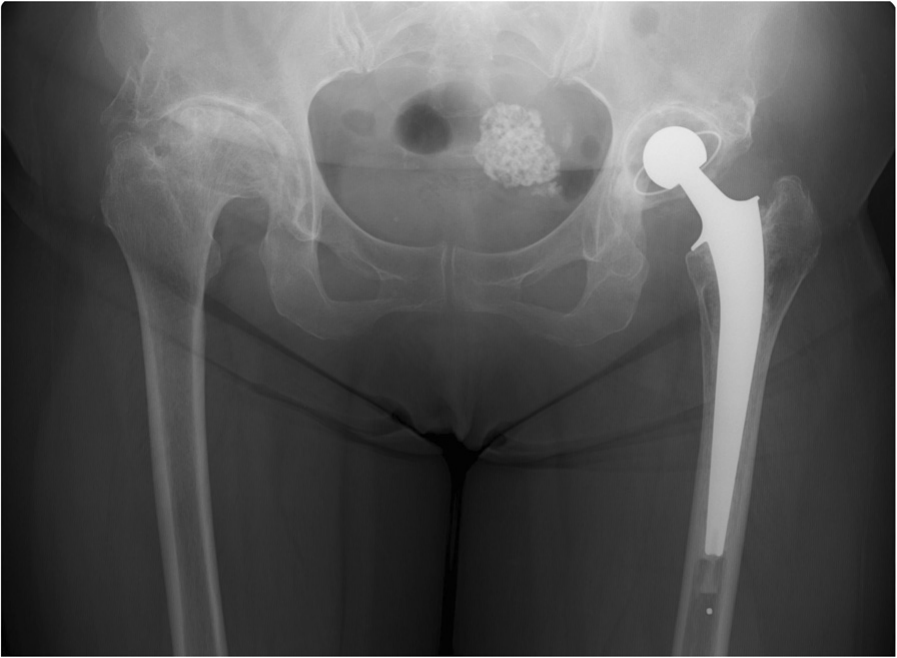
Plain radiography of the bilateral hip joints at 3 years and 6 months after surgery. Bone fusion of the periacetabular osteotomy pseudoarthrosis region is complete at 3 years and 6 months after the surgery. No loosening of the acetabular cup or stem was observed at 4.5 years after surgery

The use of a wheelchair was permitted after drain removal at 2 days after surgery, and partial and full weight bearing was permitted at 3 and 6 weeks after surgery, respectively. The postoperative course was generally favorable, and bone fusion of the PAO segment was complete at 3 years and 6 months after surgery (Fig. 3). The patient’s modified Harris hip score was 43 before the surgery, and had improved to 90 at the final follow-up.

## Discussion

We encountered a patient who underwent right PAO at the age of 52 years and left PAO at 55 years; she subsequently developed pseudoarthrosis of the left PAO segment and was successfully treated via modified THA. To the best of our knowledge, there is only one reported case of pseudoarthrosis after iliac osteotomy in a patient who was treated via triple pelvic osteotomy [6], and no reports have described treatment for pseudoarthrosis of the PAO segment via modified THA.

Patients who undergo PAO before they are 50-years old may experience favorable joint remodeling and clinical outcomes for >10 years after surgery if suitable conditions exist (for example, articular congruence). However, patients who are ≥50-years old have a poor prognosis after bilateral PAO [2–5]. In the present case, our patient was >50 years old, obese and had bilateral osteoarthritis, which are all risk factors for a poor outcome after PAO. However, the present case is unusual, as no previous cases have exhibited advanced and aggravated arthrosis with pseudoarthrosis of the iliac osteotomy segment. We hypothesize that her pseudoarthrosis may have been caused by obesity and bilateral PAO. Therefore, we suggest that bilateral PAO should be carefully selected in patients who are obese.

Although the reported outcomes for THA after PAO are favorable [7, 8], this procedure is generally more difficult than primary THA. This difficulty is probably related to various issues that may develop in the hip joint after PAO, which include acetabular osteosclerosis, anterior spur formation, acetabular inner and posterior wall bone defects, or poor articular congruence-induced femoral head deformity. Moreover, our patient only had fibrous fusion of the PAO segment (rather than osseous fusion), which created concerns that the surgery might exacerbate osteotomy segment instability and cause poor early fixation of the acetabular cup. Therefore, we chose to perform a modified THA to reacquire stability of her distal iliac osteotomy segment. There are two possible methods for this procedure. The first method is exposure of the region between the ilium and acetabular osteotomy segment, and curettage of the pseudoarthrosis region with refixation of the osteotomy segment. The second method is fixation with an absorbable screw and bone pegs, without exposing the pseudoarthrosis region, which we selected for our patient. Although the appropriate approach remains controversial, we selected the second method because we observed fibrous fusion of the pseudoarthrosis region and dissection of the affected region can increase surgical stress. Thus, we fixed the PAO mobile bone segment with one absorbable screw, and two bone pegs (prepared using autologous and allogeneic bone) were transplanted to achieve bone fusion. We also used drilling to expose the native bone and achieve favorable fixation of the cup. Cement fixation was used because the distal osteotomy segment was sclerotic, which precluded bone ingrowth fixation of the acetabular cup. We believe that this technique was successful as complete bone fusion of the PAO pseudoarthrosis region was observed at 3 years and 6 months after surgery.

In this case, no loosening of the acetabular cup and stem was observed at 4.5 years after surgery, and we successfully treated the patient via modified THA. However, placement of the cemented cup superiorly at a high hip center can affect the long-term outcome of the case [9, 10]. Several biomechanical studies have demonstrated that superolateral placement of the hip center may lead to increased moments and forces across the hip, however, superior only displacement of the hip center did not adversely affect the forces about the hip [11]. Therefore, we believe that additional follow-up may be needed to confirm the long-term outcome of the modified THA.

## Conclusions

We successfully used modified THA to treat a patient with osteoarthritis of the hip and pseudoarthrosis of the PAO segment; this technique achieved bone fusion of the pseudoarthrosis region and a favorable postoperative outcome.

## Consent

Written informed consent was obtained from the patient for publication of this case report and accompanying images. A copy of the written consent is available for review by the Editor-in-Chief of this journal.
